# Cyclin D1 Stability Is Partly Controlled by *O*-GlcNAcylation

**DOI:** 10.3389/fendo.2019.00106

**Published:** 2019-02-22

**Authors:** Louis Masclef, Vanessa Dehennaut, Marlène Mortuaire, Céline Schulz, Maïté Leturcq, Tony Lefebvre, Anne-Sophie Vercoutter-Edouart

**Affiliations:** ^1^Université de Lille, CNRS, UMR 8576, UGSF, Unité de Glycobiologie Structurale et Fonctionnelle, Lille, France; ^2^Institut Pasteur de Lille, Université de Lille, CNRS, UMR 8161, M3T: Mechanisms of Tumorigenesis and Targeted Therapies, Lille, France

**Keywords:** cyclin D, *O*-GlcNAc, stability, ubiquitination, cell cycle

## Abstract

Cyclin D1 is the regulatory partner of the cyclin-dependent kinases (CDKs) CDK4 or CDK6. Once associated and activated, the cyclin D1/CDK complexes drive the cell cycle entry and G1 phase progression in response to extracellular signals. To ensure their timely and accurate activation during cell cycle progression, cyclin D1 turnover is finely controlled by phosphorylation and ubiquitination. Here we show that the dynamic and reversible *O-*linked β-N-Acetyl-glucosaminylation (*O*-GlcNAcylation) regulates also cyclin D1 half-life. High *O*-GlcNAc levels increase the stability of cyclin D1, while reduction of *O*-GlcNAcylation strongly decreases it. Moreover, elevation of *O*-GlcNAc levels through *O*-GlcNAcase (OGA) inhibition significantly slows down the ubiquitination of cyclin D1. Finally, biochemical and cell imaging experiments in human cancer cells reveal that the *O*-GlcNAc transferase (OGT) binds to and glycosylates cyclin D1. We conclude that *O*-GlcNAcylation promotes the stability of cyclin D1 through modulating its ubiquitination.

## Introduction

In mammalian cells, progression from G1 to S phase is controlled by the cyclin D-Cyclin-dependent kinase (CDK) 4/6 complexes, in which cyclin D1 is the regulatory subunit and CDK4-6 the catalytic subunit. These complexes initiate the phosphorylation-dependent inactivation of the retinoblastoma protein pRb, promoting the E2F-dependent gene transcription, including cyclin E. Complete inhibition of pRb is then achieved by the cyclin E-CDK2 complex, allowing the expression of target genes necessary for S phase entry. To trigger a timely activation of CDK4/6 in G1 phase, cyclin D1 steady-state level is highly regulated throughout the cell cycle (1, 2). Mitogenic stimulation of quiescent cells induces a dramatic increase of cyclin D1 mRNA and protein expression, mainly through Ras/MAPK and PI3K/Akt signaling pathways which activate many transcription factors including c-Fos/c-Jun, NF-κb and β-catenin/TCF-4 (2–5). Cyclin D1-CDK4/6 complexes are then phosphorylated by CDK-activating kinase (CAK) leading to their activation and translocation to the nucleus (6, 7). Cyclin D1 has a short half-life of around 30 min, its degradation being mediated through the ubiquitin-dependent proteasomal pathway. Once cells progress in S phase, GSK-3β is translocated into the nucleus where it phosphorylates cyclin D1 at Thr286 located in a PEST C-terminal sequence (8, 9). This phosphorylation induces the export of cyclin D1 out of nucleus through a CRM1-dependent mechanism (10, 11). Additionally, phosphorylation at Thr288 by the arginine-directed serine/threonine kinase Mirk/dyrk1B induces a more rapid turnover of cyclin D1 (12). Cyclin D1 is then targeted to the SCF (SKP1 (S-phase-kinase associated protein 1), Cullin-1/Cdc53, F-Box protein) E3 ubiquitin ligase complex that promotes its polyubiquitination, leading to its subsequent proteasomal degradation (13–16). The ubiquitin-dependent proteolysis of cyclin D1 is antagonized by the deubiquitinating enzyme USP2, thus increasing cyclin D1 half-life (17). More recently, USP22 has also been identified as a deubiquitinase targeting cyclin D1 (18). Pharmacological inhibition of USP2 accelerates cyclin D1 degradation and leads to cell cycle arrest in several cancer cell lines among which the HCT116 colon cancer cell line and MCF7 breast cancer cell line (19). Importantly, deregulation of cyclin D1 stability contributes to its oncogenic potential. The absence of Thr286 in the natural alternative splicing variant cyclin D1b or the mutation T286A induce the nuclear accumulation of the cyclin, due to a defect in the phosphorylation-mediated nuclear export, and increase its oncogenicity in murine fibroblasts (10, 20). Additionally, mutations in Fbxo4 in esophageal tumors affect the E3 ligase activity and lead to overexpression of cyclin D1 (21).

Beside phosphorylation, *O*-linked β-N-acetylglucosaminylation or *O-*GlcNAcylation of proteins takes part in cell cycle regulation (22). The cycling of *O-*GlcNAcylation is controlled solely by two enzymes which are localized in the nucleus and cytoplasm. The *O*-GlcNAc Transferase (OGT) catalyzes the transfer of the GlcNAc moiety from the nucleotide sugar UDP-GlcNAc onto the hydroxyl group of Ser/Thr of resident proteins of the nucleus, cytoplasm and mitochondria, while the *O*-GlcNAc hydrolase (OGA) reverses the reaction. *O*-GlcNAcylation regulates various biological functions, such as protein interactions, protein stability, subcellular localization, and enzyme activity (23–25). Interestingly, a reciprocal relationship between *O*-GlcNAc and phosphorylation has been described for many proteins such as the transcription factors c-Myc and delta-lactoferrin (26, 27), and the signaling kinase Akt (28). Importantly, overexpression of OGT and hyper *O*-GlcNAcylation of proteins have been emerging for the last decade as a landmark of cancer cells (29, 30). Deciphering the molecular mechanisms that are regulated by *O*-GlcNAc cycling is thus crucial to better understand how *O*-GlcNAcylation impacts cancer cells properties. In mammalian cells, *O*-GlcNAc levels are regulated in a cell cycle-dependent manner and dynamics of *O*-GlcNAcylation is important for the correct progression of cell cycle (31–33). Inhibition of OGT catalytic activity or down-regulation of its expression hindered serum-induced cyclin D protein expression and cell cycle entry (34). Serum stimulation of *Ogt*-deficient mouse embryonic fibroblasts failed to enhance the protein levels of c-Fos, c-Jun, and c-Myc, whereas level of the inhibitor p27^KIP1^ was highly increased (35). The stability of p27^KIP1^ was also increased in human cancer cells in which OGT was knockdown, concomitantly to a significant decrease of proliferation (36, 37). Moreover, impairment of *O*-GlcNAc cycling altered the protein levels of cyclins D and E in both OGT- and OGA- overexpressing cells (31), while shOGT decreased cyclin D expression in pancreatic cancer cells (38). Cyclin D expression was also slightly altered in stable OGA knockdown cells (39).

Despite mounting evidence of a link between cyclin D1 steady-state level and *O*-GlcNAc homeostasis, the underlying molecular mechanisms are still unclear. Therefore, we investigated how *O*-GlcNAcylation could regulate cyclin D1 expression and whether cyclin D1 could be *O*-GlcNAcylated. Here, we demonstrate that cyclin D1 stability is positively regulated with *O*-GlcNAcylation levels in human cancer cells. High *O*-GlcNAc conditions perturb the polyubiquitination of cyclin D1, thus slowing down its degradation rate. This study also reveals that OGT binds to cyclin D1, mostly in the nucleus and in a cell cycle-dependent manner. Finally, we describe for the first time the *O*-GlcNAcylation of cyclin D1. Altogether, this work highlights a novel post-translational modification of cyclin D1 that helps its stability. It also provides novel molecular insights into the role of *O*-GlcNAc cycling in cell cycle regulation.

## Materials and Methods

### Cell Culture

HEK293T, MCF7, and HCT116 cells were cultured at 37°C in DMEM supplemented with 10% (v/v) fetal calf serum (Lonza, Ozyme, France), in a humidified atmosphere enriched with 5% (v/v) CO_2_.

### Plasmids, siRNA, and Inhibitors

pTAT-cyclin D1 expression vector was obtained from Dr. B. Sola (EA4652, Université de Caen Normandie, Caen, France). The FLAG-Cyclin D1 vector was generated by cloning cyclin D1 cDNA into pcDNA3.1 using Kpn1 and EcoRI restriction enzymes. HA-OGT was obtained from Dr. T. Issad (Inserm, U1016, Institut Cochin, Paris, France), pCMV-Myc-Cyclin D was kindly provided by Dr. X. Ye (Institute of Microbiology, Chinese Academy of Sciences, Beijing, China) (40). The Ub-HA expression vector was a gift from Dr. C. Couturier (Institut Pasteur de Lille, U1177, Lille, France). Control siRNA (SIRNA UNIV NEGATIVE CONTROL 1, SIC001) and OGT siRNA (GGAGGCUAUUCGAAUCAGU[dT][dT] and ACUGAUUCGAAUAGCCU-CC[dT][dT]) were purchased from Sigma-Aldrich (La Verpillière, France) and siGENOME Human MGEA5 (10724) siRNA-SMARTpool was purchased from Dharmacon (GE Healthcare Europe GmbH, Velizy-Villacoublay).

The OGT inhibitor Acetyl-5S-GlcNAc (Ac-5S-G, 100 mM stock solution in DMSO) was a kind gift of Dr. G.W. Hart (The Johns Hopkins University School of Medicine, Baltimore, USA). The OGA inhibitor Thiamet G (ThG, 10 mM stock solution in DMSO) and cycloheximide (CHX, 5 mg/ml stock solution in DMSO) were from Sigma-Aldrich. The proteasome inhibitor MG132 (20 mM stock solution in DMSO) was purchased from Cayman Chemical (V.W.R., Fontenay-sous-Bois, France). For controls, vehicle (DMSO) was added at the same final dilution.

### Antibodies

Antibodies against cyclin D1 (DCS-6, sc-20044; A12, sc-8396), HA-tag (sc-805), GAPDH (sc-47724), beta-actin (sc-1616) and normal rabbit or mouse IgG were from Santa Cruz (Heidelberg, Germany). Antibodies against FLAG-tag (M2) and OGT (Ti-14 and DM-17) were purchased from Sigma-Aldrich. Rabbit polyclonal anti-MGEA5 antibody (anti-OGA, EPR7154B) was from Abcam (Cambridge, United Kingdom). Anti-*O-*Linked N-Acetylglucosamine monoclonal antibody (RL2) was purchased from Thermo Scientific (V.W.R.). Secondary rabbit or mouse IgG HRP-linked antibodies were from GE Healthcare (V.W.R.). Anti-rabbit IgG Alexa Fluor 488 and anti-mouse IgG Alexa Fluor 568 (ThermoScientific, Fisher Scientific, France) were used for immunofluorescence.

### Transfection

HEK293T (40 × 10^3^ cells per well in 12-wells plate; 8 × 10^5^ cells per 100 mm-Petri dish) and HCT116 (8 × 10^5^ cells per 100 mm-Petri dish) cells were grown for 24 h. Prior to transfection, plasmids were diluted in Ultra-MEM (Lonza, Ozyme) and mixed for 20 min with Lipofectamine 2000 (Life Technologies, Fisher Scientific, Illkirch, France), according to manufacturer's instructions. The DNA-lipid complex (500 ng DNA/well in 12-well plates, 1.25 μg of each vector for co-transfection or 2 μg DNA per 100 mm-dish for HEK293T) was added in fresh medium and transfected cells were analyzed 48 h later. The various treatments with the inhibitors were done as indicated in the text and figure legends. Reverse transfection of siRNA was performed in complete medium (2 × 10^5^ or 3.75 × 10^5^ cells in 6-well plates for HEK 293T and MCF7 cells respectively; 6 × 10^5^ or 1 × 10^6^ in 100-mm dishes for HEK 293T and MCF7 cells, respectively), as previously described (41). Transfected cells were harvested 72 h later.

### Quantitative RT-PCR

RNA was isolated using Nucleospin® RNA mini spin kit (Macherey-Nagel) according to the manufacturer's instructions. 1 μg of total RNA was reverse transcribed using random primers and MultiScribe™ reverse transcriptase (Applied Biosystems). Real-time PCR analysis was performed by Power SYBR Green (Applied Biosystems) in a MX3005P fluorescence temperature cycler (Stratagene) according to the manufacturer's instructions. The sequences of the primers used for the RT-qPCR analyses were as follows: OGT forward 5′-TGG CTT CAG GAA GGC TAT TG-3′ and reverse 5′-CAA GTC TTT TGG ATG TTC ATA TG-3′; cyclin D1 forward 5′-CAT CTA CAC CGA CAA CTC CAT CC-3′ and reverse 5′-TGT TCA ATG AAA TCG TGC GG-3′; RPLP0 (ribosomal protein large subunit P0) forward 5′-GTG ATG TGC AGC TGA TCA AGA-3′ and reverse 5′-GAT GAC CAG CCC AAA GGA GA-3′.

### Cell Treatment and Synchronization

HCT116 cells (40 × 10^3^) and MCF7 cells (55 × 10^3^) were cultured in 12-well plates for 2 days. Cells were treated overnight with Ac-5S-G (50 or 100 μM) or ThG (1 μM). Then CHX (50 μg/ml) or MG132 (20 μM) were used in time-course experiments. For serum starvation time-course experiments, OGT or OGA inhibitors were added in the complete medium 2 h before starvation and during serum starvation in serum-free medium.

### Cell Cycle Analysis

Distribution of cells in G0/G1, S, and G2/M was determined by DNA staining with propidium iodide as previously described (33).

### sWGA Lectin Affinity Chromatography

HCT116 cells and cyclin D1-FLAG-transfected HEK293 T cells were treated overnight with vehicle (DMSO) or ThG (1 μM) before lysis in RIPA (50 mM Tris pH 7.5, 150 mM NaCl, 1% NP-40, 0.25% sodium deoxycholate and 0.1% SDS). *O*-GlcNAc-modified proteins were enriched using the non-reducing terminal GlcNAc-specific lectin succinylated wheat germ agglutinin (sWGA) immobilized on agarose beads (Vector Laboratories, Clinisciences, Nanterre, France). Lysates (4 mg, 1 mg/ml) were incubated overnight at 4°C with 200 μl of sWGA-beads. Beads were centrifuged at 1,000 × g for 3 min, and then washed under vigorous stirring twice with 2 ml RIPA-0.1% SDS buffer and then three times with 2 ml RIPA-0.2% SDS buffer. Finally, beads were re-suspended in Laemmli buffer and stirred for 10 min using a vortex mixer before heating at 95°C for 7 min. Negative control was performed by adding 0.5 M free GlcNAc in the cell lysate before incubation with sWGA-beads. Eluted proteins were loaded on 10% SDS-PAGE to perform Western-blot analysis.

### Enzymatic Labeling and Click Chemistry

Whole cell lysate of cyclin D1-FLAG-transfected HEK293 T cells (200 μg) and α-crystallin (20 μg) as positive control were processed using the click-It™ *O*-GlcNAc enzymatic labeling and click-It™ biotin glycoprotein D kits (Invitrogen, Thermo Fisher scientific), according to manufacturer's instruction. Enrichment of biotinylated proteins was performed as previously described (42).

### Immunoprecipitation

Cells were rinsed twice in cold-PBS, then lysed on ice for 20 min in 400 μl RIPA buffer (without SDS for co-immunoprecipitation) supplemented with a protease inhibitor cocktail (Sigma-Aldrich), 10 mM Sodium Fluoride and 1 mM sodium orthovanadate. Cell extracts were centrifuged at 20,000 × g for 15 min at 4°C and supernatants were kept at −20°C. For immunoprecipitation, one mg of total proteins for transfected cells and 2 mg of total proteins for non-transfected cells (volume adjusted for a final concentration of 2 mg/ml) were pre-cleared with protein A/protein G-sepharose beads (20 μl/mg) (GE Healthcare, V.W.R.) for 1 h at 4°C. After centrifugation (1,200 × g, 5 min, 4°C), the supernatant was incubated overnight with the primary antibody (2 μg antibody per mg of protein) under gentle agitation. Protein A/G beads (30 μl/mg) were added for 1 h at 4°C before centrifugation and washes of the beads (three times in 1 ml RIPA and once in modified RIPA containing 450 mM NaCl, 5 min each). Beads were heated at 95°C for 7 min in 25 μl Laemmli buffer before SDS-PAGE.

### Western Blot Analysis

Proteins were separated by SDS-PAGE and transferred onto nitrocellulose membrane (Hybond-C Extra, GE Healthcare) at 200 mA for 2 h. Membranes were blocked with 5% (w/v) nonfat dry milk in TBST (15 mM Tris pH 8, 140 mM NaCl, 0.05% (v/v) Tween-20) at room temperature (R.T.) for 45 min, then incubated overnight at 4°C with the primary antibody diluted in the blocking solution [1:1,000 except for anti-OGT and anti-GAPDH (1:2,000), anti-*O*-GlcNAc (1:4,000) and anti-OGA (1:10,000)]. Membranes were washed with TBST (3 × 7 min) and incubated with secondary HRP-linked antibodies (1:15,000 in TBST) for 1 h at R.T. After three washes in TBST, detection was performed using chemiluminescence (ECL Prime, GE Healthcare; Supersignal West Femto Max., Thermo Scientific) on a CCD camera (Fusion Solo, Vilber Lourmat, France). Membranes were stripped in stripping buffer (ST010, GenBio, Euromedex, Souffelweyersheim, France) for 10 min and extensively washed in TBST before incubation with other antibodies. Quantification of protein expression levels was calculated by densitometry using the ImageJ® software. Student's *t*-test (Excel) was used for statistical analysis; *p*-values were calculated and reported accordingly (^*^p < 0.1, ^**^p < 0.05, ^***^p < 0.005).

### Indirect Immunofluorescence and Proximity Ligation Assay

Cells were grown in 6-well plates on glass coverslips (20 × 10^4^ cells/well) for 2 days. Cells were then starved for 24 h (in DMEM or DMEM-0.5% (v/v) FCS for MCF7 cells) before serum stimulation for the indicated time periods. Immunofluorescence experiments were done as previously described (33). Briefly, after fixation of cells in 4% (w/v) PAF (30 min at R.T.) and permeabilization (0.5% Triton X-100 in PBS, 20 min at R.T.), coverslips were incubated with the blocking buffer containing 2% (v/v) FCS, 2% (w/v) bovine serum albumin, 0.2% (w/v) gelatin in PBS (1 h at R.T.) before incubation with primary antibodies against cyclin D1 (A12) and OGT (Ti-14) (1:100 in blocking buffer, overnight at 4°C) and Alexa Fluor conjugated secondary antibodies (1:600 in blocking buffer, 1 h at R.T). For the Proximity ligation assay (Duolink® *in situ* kit, Sigma-Aldrich), primary antibodies were incubated on fixed cells in the blocking buffer provided in the kit (1:100). Manufacturer's instructions were followed for the incubation with minus and plus probes, the ligation and amplification (120 min, 37°C) steps (Duolink® *in Situ* Detection Reagents Green, Sigma-Aldrich). After mounting coverslips in fluorescence mounting medium (DAKO, Agilent Technologies France, Les Ulis, France), images were acquired using an inverted Zeiss LSM700 confocal microscope with a 40x oil immersion lens at R.T. and data were collected with the ZEN 2010 software (Zeiss, Oberkochen, Germany). Images from PLA were processed with ImageJ® using a home-made plugin developed by TISBio to detect and quantify the nuclear fluorescent dots in labeled cells. Scatter dot plot (median with interquartile range) showing nuclear fluorescence intensity quantified in each cell (two captured images per condition) and statistical analysis were obtained using GraphPad Prism software (one-way ANOVA test, ^***^p < 0.0001, ^**^p < 0.005, ^*^p < 0.05).

## Results

### Perturbation of *O*-GlcNAc Cycling Modifies the Half-Life of Cyclin D1

**Figure 1:**
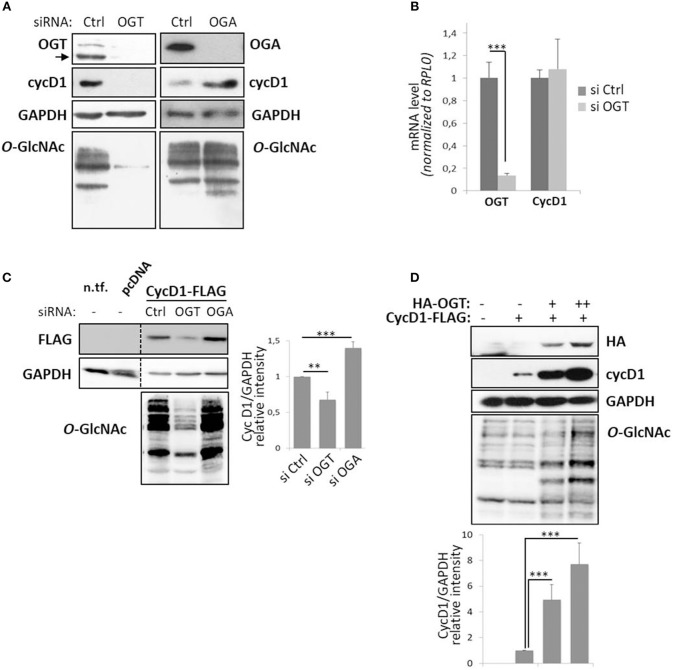
Perturbation of *O*-GlcNAc cycling affects cyclin D1 protein expression level. MCF7 cells were transfected for 72 h with siRNA (Control (Ctrl), OGT, or OGA) and harvested to get protein lysates or mRNA samples. **(A)** Western-blotting analysis following siRNA show depletion of targeted proteins and *O*-GlcNAcylated proteins with concomitant variation in cyclin D1 (cycD1) level. **(B)** OGT and cycD1 mRNA levels were quantified by qPCR using RPLP0 as internal control. Results correspond to the mean value ± s.d. of three independent experiments (***p < 0.005). **(C)** HEK293T cells were seeded in 12-well plates with siRNA (Ctrl, OGT, or OGA) for 24 h and then transfected with pcDNA3.1 or CycD1-FLAG (100 ng). Cells were lysed 2 days later (three independent experiments). Lysate from non-transfected HEK293T cells (n.tf.) was also loaded on the same gel. **(D)** HEK293T cells were transfected in 12-well plates for 48 h with CycD1-FLAG (500 ng) and OGT-HA (500 ng or 1 μg) and then lysed in Laemmli buffer (two independent experiments). **(C,D)** The cellular lysates were analyzed by Western blot using specific antibodies. Histograms represent the relative intensity of cyclin D1 expression levels normalized to GAPDH levels. Statistical analyses were performed by Student's *t*-test (^***^p < 0.005, ^**^p < 0.05).

To examine the effect of perturbation of *O*-GlcNAc cycling on cyclin D1 expression, MCF7 cells were depleted of their OGT or OGA content by using small interfering RNA (siRNA) strategy. As previously reported, the level of cyclin D1 increased when OGA was silenced and, conversely, decreased when OGT was knocked-down (Figure 1A) (34, 38, 39). On the other hand, the mRNA levels of cyclin D1 did not change in OGT depleted condition (siOGT) compared with the control (siCtrl), while the OGT mRNA levels dramatically decreased in siOGT-treated cells (Figure 1B). This result demonstrates that the decrease of cyclin D1 protein level in siOGT cells did not result from a diminution of its transcription in MCF7 cells. Similar results were obtained when cyclin D1-FLAG was overexpressed in siOGT- or siOGA-treated HEK293T cells. The knock-down of OGT led to a 40%-decrease of cyclin D1-FLAG steady-state level, whereas elevation of *O*-GlcNAcylation in siOGA-treated cells led to a 30%-increase of the protein (Figure 1C). Lastly, when cyclin D1-FLAG was overexpressed in HEK293T cells with increasing amount of the plasmid encoding HA-OGT, we observed a positive correlation between the expression of HA-OGT and cyclin D1 protein levels (Figure 1D).

**Figure 2:**
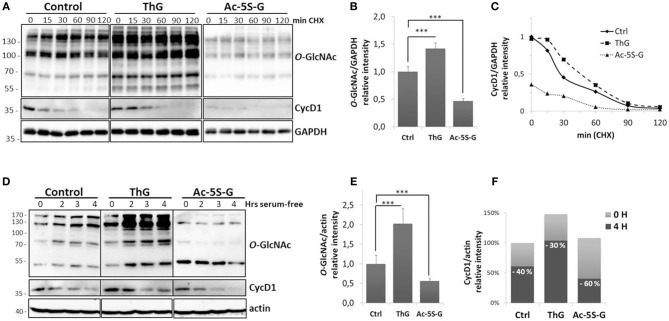
Turnover of cyclin D1 is dependent to *O-*GlcNAc homeostasis. **(A–C)** MCF7 cells were treated overnight with Ac-5S-G (100 μM) or ThG (1 μM). Then CHX (50 μg/ml) was added into the medium before cell lysis at indicated times. **(A)**, Western-blots show the levels of *O*-GlcNAcylated proteins and cyclin D1. GAPDH served as a control for equal loading. **(B)** The histogram represents the relative quantification of *O*-GlcNAc levels in treated cells compared with control cells. **(C)** Graph shows the ratios of cyclin D1 to GAPDH. Representative results of three independent experiments. **(D–F)** HCT116 cells were pretreated 2 h with vehicle (DMSO, Control), ThG (1 μM), or Ac-5S-G (50 μM). Cells were starved in serum-free medium for the indicated times prior to cell lysis. **(D)** Western-blot show the levels of *O*-GlcNAcylated proteins and cyclin D1. Actin served as a control for equal loading. **(E)** The histogram represents the relative quantification of *O*-GlcNAc levels in treated cells compared with control cells. **(F)** The histogram represents the relative intensity of cyclin D1 levels normalized to actin levels (100% is the normalized level of cyclin D1 in the control cells at T0). On each histogram, the percentage of the decrease of cyclin D1 level after 4 h of serum starvation is indicated. Results are representative of two independent experiments. Statistical analysis in (**B**,**E)** was performed by Student's *t*-test (^***^p < 0.005).

In proliferating cells, the level of cyclin D1 is tightly controlled by the balance between the increase of its expression induced by the activation of mitogenic signaling pathways and its ubiquitin-mediated degradation (2, 5). To monitor the effect of *O*-GlcNAc cycling impairment on cyclin D1 half-life, MCF7 cells were treated overnight with Acetyl-5S-GlcNAc (Ac-5S-G) or Thiamet G (ThG) to inhibit, respectively the OGT and OGA catalytic activities prior to treatment with the protein synthesis inhibitor CHX. Efficiency of OGT or OGA inhibition was confirmed by Western-blot analysis (Figure 2A). We observed a significant increase of *O*-GlcNAcylation in ThG-treated cells and conversely a strong decrease of *O*-GlcNAcylation levels in cells treated with Ac-5S-G (Figure 2B). We showed by Western blot that the time-dependent decrease of cyclin D1 level was slowed down by ThG treatment compared with control cells (Figure 2A): the half-life time of cyclin D1 was 30 min in control cells and reached 50 min in ThG treated cells (Figure 2C). In Ac-5S-G treated cells, the initial level of cyclin D1 was decreased by more than a half compared to control cells. However, the time-course degradation of cyclin D1 was slowed down, since 50% of cyclin D1 protein steady-state level was reached after 40 min of CHX treatment (Figure 2C).

Downregulation of cyclin D1 upon serum deprivation contributes to cell cycle exit (43). To test whether perturbation of *O*-GlcNAc cycling could affect the decrease of cyclin D1 level induced by serum starvation, HCT116 cells were treated by ThG or Ac-5S-G 2 h prior to serum starvation and cyclin D1 level was monitored by Western-Blot. Efficiency of inhibitors was monitored by Western-blot against *O*-GlcNAcylated proteins (Figures 2D,E). Upon mitogen withdrawal, cyclin D1 levels decreased in a time-dependent manner both in control and treated cells (Figure 2D). Four hours after serum starvation, we observed a 40% decrease of cyclin D1 level in control cells (Figure 2F). This decrease was less pronounced in ThG-treated cells in which a 30% decrease of cyclin D1 was observed 4 h following serum starvation. In contrast, reduction of *O*-GlcNAcylation by inhibiting OGT accelerated the degradation of cyclin D1 since 60% of the protein was lost after 4 h of starvation (Figure 2F). These results corroborate our previous observations regarding treatment of cells with CHX, and suggest that *O*-GlcNAc cycling participates in the regulation of cyclin D1 protein level.

### OGT Interacts With and Glycosylates Cyclin D1

**Figure 3:**
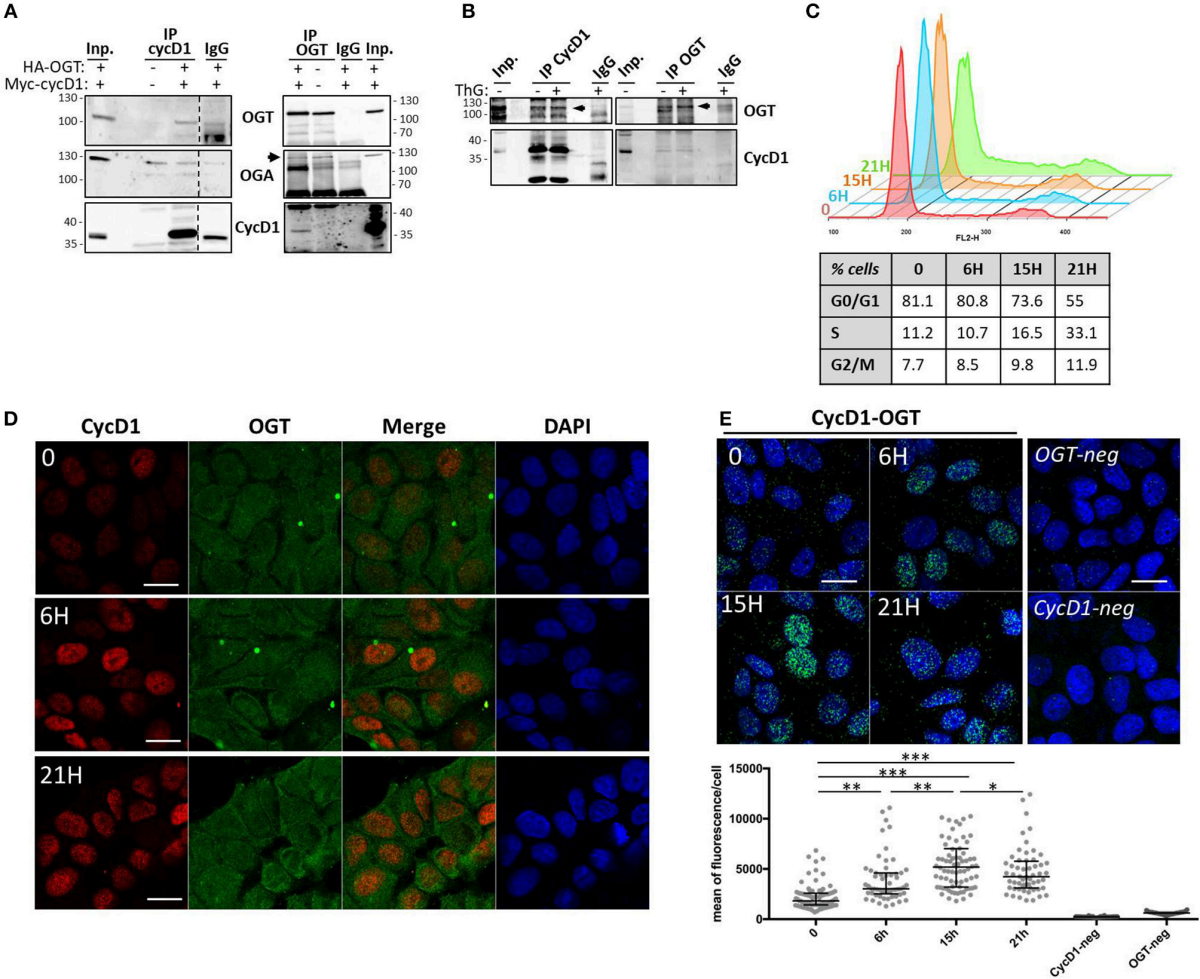
OGT interacts with cyclin D1. **(A)** HEK293T cells were co-transfected with HA-OGT and Myc-cyclin D1 plasmids for 48 h before cell lysis. Cyclin D1 and OGT were immunoprecipitated and Western-blot analysis were performed to detect OGT, OGA and cyclin D1. Negative controls of IP were done using non-relevant IgG (IgG). **(B)** HCT116 cells were treated overnight with DMSO (-) or ThG (1 μM) (+) before cell lysis in RIPA buffer and immunoprecipitation of endogenous cyclin D1 and OGT. Western-blot analysis of immunoprecipitates (IP) and Input (Inp.) using OGT and cycD1 antibodies. **(C**–**E)** Quiescent MCF7 cells (0) were stimulated with serum for the indicated times. **(C)** Cell cycle profile was monitored by flow cytometry after staining with propidium iodide. The percentage of cells in each phase is reported, according to serum release for each time point. **(D)** Cyclin D1 and OGT were detected in synchronized MCF7 cells by confocal indirect immunofluorescence using specific antibodies. Nuclei were counterstained with DAPI. Scale bar, 20 μM. **(E)** The interaction between OGT and cyclin D1 was detected using *in situ* PLA and immunofluorescent confocal microscopy. Nuclei were stained with DAPI. Pictures are the merge of PLA signal (AlexaFluo488) and DAPI channels. Quantification of PLA is presented as scatter dot plot; each dot represents the mean of PLA fluorescence intensity in the nucleus of a single cell. Bars represents the median with interquartile range for each experience (one-way ANOVA test, ^***^p < 0.0001, ^**^p < 0.005, ^*^p < 0.05). Scale bar, 20 μM.

One of the well-established roles of OGT is to interact with and protect its targets from proteasomal degradation (44–48). To first examine whether OGT binds to cyclin D1, we performed co-immunoprecipitation experiments in HEK293T cells ectopically co-expressing cyclin D1-Myc and HA-OGT. As shown in Figure 3A, a band corresponding to OGT was detected in cyclin D1 co-immunoprecipitates when both plasmids were co-transfected in cells (IP cycD1, *lane 3*). Conversely, a small fraction of cyclin D1 was detected when OGT was immunoprecipitated (Figure 3A, IP OGT, *lane 1*). Cyclin D1 was undetectable in non-transfected cells although endogenous OGT was successfully immunoprecipitated (Figure 3A, IP OGT, *lane 2*). A specific band corresponding to OGA was detected after OGT immunoprecipitation (Figure 3A, IP OGT, *lanes 1- 2*) but not after cyclin D1 immunoprecipitation where we observed the same 130 kDa-band in the IgG negative control (Figure 3A, IP cycD1, *lanes 2-4*). This result suggests that cyclin D1 may directly interact with OGT, whereas OGA is present in the complex through its tight interaction with OGT, as previously described (49). We performed the same set of experiments on endogenous OGT and cyclin D1 proteins in HCT116 cells, confirming that both proteins can interact (Figure 3B). Treatment of HCT116 cells with ThG did not change the co-immunoprecipitation of cyclin D1 and OGT.

To further characterize cyclin D1/OGT interaction, we performed *in situ* PLA experiments. This approach allows gaining in sensitivity thanks to the ligation and amplification steps. For this purpose, serum-starved quiescent MCF7 cells (T0) were stimulated by addition of serum to re-enter the cell cycle. Cells were fixed in G1 phase (6 h), S phase entry (15 h) and S phase (21 h), as attested by flow cytometry (Figure 3C). First, indirect immunofluorescence experiments in synchronized MCF7 cells confirmed that cyclin D1 is translocated to the nucleus upon cell cycle entry, whereas OGT is detected in both the cytoplasm and the nucleus (Figure 3D). The PLA signal revealed that cyclin D1/OGT interaction was detectable in quiescent cells, both in the cytoplasm and the nucleus. The intensity of the PLA signal increased in the nucleus as cells progressed through G1 and entered S phase, and then slightly decreased when cells progressed through S phase (Figure 3E). Our data indicate that OGT and cyclin D1 are likely to interact in both compartments, but this interaction is mostly detected in the nucleus of G1-cells, concomitantly to the activation and nuclear translocation of cyclin D1 upon serum stimulation.

**Figure 4:**
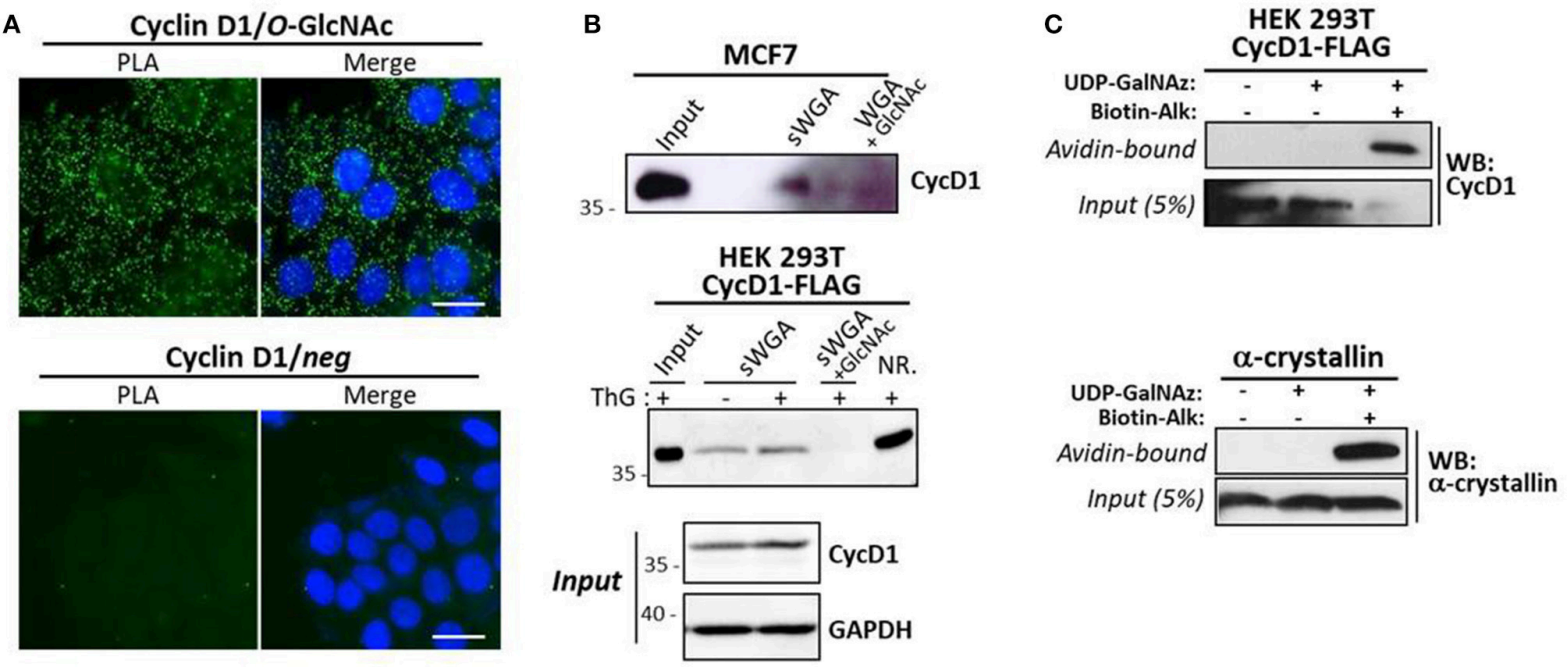
Cyclin D1 is *O*-GlcNAcylated. **(A)** *in situ* PLA experiments by using anti-cyclin D1 and anti-O-GlcNAc (RL2) antibodies on asynchronous MCF7 cells. Nuclei were stained with DAPI. PLA signal was detected by immunofluorescent confocal microscopy. Scale bar, 20 μM. **(B)** MCF7 cells were treated with MG132 (4 μM) for 2 h prior cell lysis. Cyc D1-FLAG transfected HEK293T cells were treated overnight or not with ThG (1 μM) and lysed in RIPA buffer. *O*-GlcNAcylated proteins were enriched on sWGA-agarose beads. Incubation of sWGA beads with 0.5 M GlcNAc was done as a negative control. sWGA-bound Cyclin D1 was detected by Western-blot after lectin affinity chromatography (Input, 30 μg; NR., not retained, 30 μg). **(C)** Enrichment of biotinylated proteins from CycD1-FLAG transfected HEK293T cells or purified α-crystallin after enzymatic labeling with UDP-GalNAz and click chemistry reaction using a biotin-alkyne probe. Cyclin D1 and α-crystallin were revealed by Western-Blot on avidin-bound protein fraction and Input.

We next investigated whether cyclin D1 is a direct target or not of OGT by using several experimental approaches. *In situ* PLA is widely used to study protein-protein interaction, but is also used for detecting co-/post-translational modifications such as phosphorylation and glycosylation (50, 51). Here we performed PLA using antibodies against cyclin D1 and *O*-GlcNAcylated proteins (RL2). As shown in Figure 4A, fluorescent PLA spots were visible in the cytoplasm and nucleus of asynchronous MCF7 cells, albeit at higher intensity in cytoplasm, suggesting that cyclin D1 was potentially *O*-GlcNAcylated in both compartments. However, since we cannot exclude that our PLA results could reflect the interaction of cyclin D1 with *O*-GlcNAcylated partners, we used biochemical approaches to ascertain the *O*-GlcNAcylation of cyclin D1. We first performed a succinyl-WGA affinity chromatography to enrich *O*-GlcNAcylated proteins from MCF7 cells and cyclin D1-FLAG transfected HEK293T cells (Figure 4B). As observed by Western blot analysis, a small part of endogenous cyclin D1 and overexpressed cyclin D1-FLAG was detected on sWGA-agarose beads in a specific manner (*lane sWGA*) since signals were abrogated when sWGA-beads were incubated with excess of free GlcNAc (Figure 4B). ThG treatment slightly increased the binding of cyclin D1-FLAG on sWGA-agarose beads, which may reflect the moderate increase of cyclin D1-FLAG protein level observed in ThG-treated cells compared with non-treated cells (*Input*, Figure 4B). We also performed the enzymatic labeling of *O*-GlcNAc proteins with UDP-GalNAz, followed with the click chemistry reaction using a biotin-alkyne probe as previously described (42). Negative controls were done by omitting either the two compounds, or only the biotin-alkyne probe; α-crystallin served as positive control. Biotinylated proteins were either loaded directly onto the gel (*Input* samples, 5% of the volume,) or enriched on avidin-agarose beads before SDS-PAGE (*Avidin-bound* samples). As shown by Western blot, cyclin D1 was detected in all the inputs although the signal is lower in lane 3 (Figure 4C, *Input*). It might be due to a reduction of the ligated biotin epitope accessibility by the cyclin D1 antibody compared to non-modified cyclin D1 in lanes 1 and 2. When biotinylated proteins were eluted from avidin beads, cyclin D1 was detected only in the sample in which both UDP-GalNAz and the biotin-alkyne probe were added for the click reaction, as we observed for α-crystallin. This indicates that cyclin D1 bears *O*-GlcNAc residues that could have been modified by click chemistry (Figure 4C). Of note, we added the denaturing detergent SDS in the lysis and washing buffers for the sWGA enrichment (0.2% SDS) and click chemistry (up to 1% SDS) experiments; the use of this stringent condition indicates that binding of cyclin D1 on lectin agarose-beads and avidin beads is rather hard suggesting a direct interaction through *O*-GlcNAcylated forms of the cyclin. Altogether our results show that cyclin D1 is *O*-GlcNAcylated in human cells, albeit at a very low extent.

### Elevation of *O*-GlcNAcylation Decreases Cyclin D1 Ubiquitination

**Figure 5:**
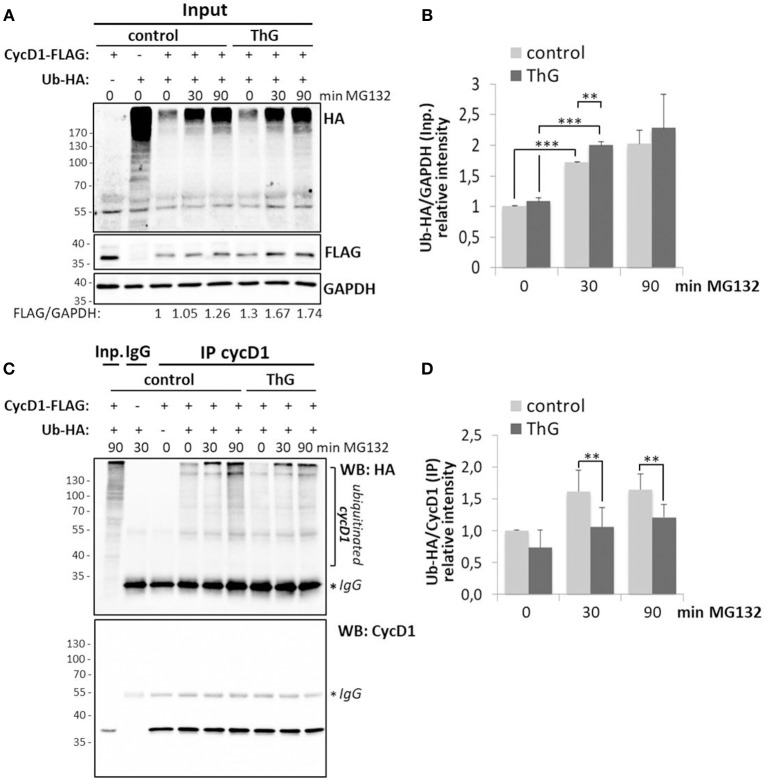
Elevation of *O*-GlcNAc levels decreases ubiquitination of cyclin D1. HEK 293T cells were transfected for 48 h with CycD1-FLAG and Ub-HA plasmids and treated or not with ThG (1 μM) overnight. MG132 (20 μM) was then added into the medium for the indicated times before cell lysis. **(A)** Input (30 μg) were revealed using anti-HA (Ub) and anti-FLAG antibodies. GAPDH was used as a loading control. **(B)** The relative ratios of ubiquitin to GAPDH levels were calculated by densitometry using Image J software (mean ± s.d., two independent experiments). **(C)** CycD1 was immunoprecipitated and Western-blot was revealed using anti-HA (Ub) and anti-CycD1 antibodies. **(D)** The ratios of the ubiquitinated cycD1 levels were calculated by densitometry using Image J® software (mean ± s.d., three independent experiments). Statistical analysis was performed by Student's *t*-test (**p < 0.05, ^***^p < 0.005).

As the protein level of cyclin D1 in proliferating cells is highly controlled by ubiquitination and since elevation of *O*-GlcNAcylation induced by OGA inhibition (ThG) or knockdown (siOGA) increases the steady-state level of cyclin D1 (Figures 1, 2), we examined the ubiquitination of cyclin D1 in presence of ThG. Cyclin D1-FLAG was ectopically expressed in HEK293T cells which were co-transfected with Ubiquitin-HA. Cyclin D1 was then immunoprecipitated in conditions of proteasome inhibition by using MG132. The ubiquitinated forms of cyclin D1 were detected by Western blot after immunoprecipitation of the cyclin. *O*-GlcNAcylation has been reported to directly modulate the proteasome activity (46, 52). Therefore, we first measured the global pattern of ubiquitinated proteins with or without ThG treatment. As shown in Figure 5A, efficiency of MG132 was attested by the increase in the amount of ubiquitinated proteins in a time-dependent manner in both conditions, and ThG treatment slightly increased the ubiquitination of proteins after 30 min of MG132, compared to control condition. However, this subtle difference was not statistically significant after 90 min of MG132 addition (Figures 5A,B). We also observed an increase of cyclin D1-FLAG levels in ThG-treated cells (Figure 5A), in agreement with our previous results (Figures 1, 2). In contrast, upon MG132 addition, ubiquitination of immunoprecipitated cyclin D1 was reduced by around 30% when cells were treated overnight with ThG, compared with control cells (Figures 5C,D). This result suggests that elevation of *O*-GlcNAc levels may protect cyclin D1 from proteasomal degradation, thus stabilizing cyclin D1 in proliferating cells.

## Discussion

In quiescent and cycling cells, cyclin D1 levels are regulated both at the transcriptional and post-translational levels in order to finely regulate cell cycle progression (1, 2). We and others have previously shown that cyclin D1 level was impaired in cells in which the dynamics of *O*-GlcNAcylation was disrupted (31, 34, 38, 39). Here, we further investigate the link between *O*-GlcNAc homeostasis and cyclin D1 expression. We show that cyclin D1 levels are positively regulated by *O*-GlcNAcylation (Figure 1). Elevation of *O*-GlcNAcylation through silencing of OGA or overexpression of OGT increases cyclin D1 steady-state levels. Conversely, decrease of *O*-GlcNAcylation by downregulation of OGT strongly reduces cyclin D1 protein level, independently of its mRNA levels (Figure 1), indicating that the regulation of cyclin D1 by *O*-GlcNAcylation is unlikely to occur at a transcriptional level. Therefore, we next examined whether dynamics of *O*-GlcNAc could regulate the degradation rate of cyclin D1. Cyclin D1 is a highly labile protein whose degradation is mainly driven by the ubiquitin-dependent proteasomal pathway (2, 8, 9, 15, 17–19). We hypothesized that increase of *O*-GlcNAcylation stabilizes cyclin D1 by interfering with its degradation. We examined the decline of cyclin D1 protein levels either by blocking protein synthesis by CHX or upon serum starvation, in high or low *O*-GlcNAc conditions. We show that increase of *O*-GlcNAc levels by OGA inhibition tends to delay the degradation rate of cyclin D1 in both experiments, while decreasing *O*-GlcNAcylation tends to accelerate it following mitogen deprivation (Figure 2). Once exported in the cytoplasm, cyclin D1 is polyubiquitinated prior to proteasomal degradation (9, 13, 14). We then determined if the stabilizing effect of ThG on cyclin D1 protein level was a consequence to a reduction of its ubiquitination. Our results confirmed that increasing *O*-GlcNAc levels reduces the accumulation of polyubiquitinated cyclin D1 (Figure 5). All these results taken together indicate that *O*-GlcNAcylation is able to slow down the ubiquitination of cyclin D1, which consequently delays its proteasomal proteolysis and reduces its turnover.

Several proteins harboring a short half-life are protected by *O*-GlcNAcylation, as described for p53, β-catenin and FOXM1 (44–48). Therefore, we investigated whether homeostasis of *O*-GlcNAcylation could regulate cyclin D1 turnover through a direct effect of OGT on the cyclin. Our results show for the first time that cyclin D1 binds to and is glycosylated by OGT. Indeed co-immunoprecipitation and PLA experiments highlight that cyclin D1 interacts with OGT in three different human cell lines (MCF7, HCT116, and HEK293T) (Figure 3). Our data strengthen the large-scale analysis of cyclin D1 interactome, in which OGT belongs to the top 30 cyclin D1-interacting proteins in MCF7 cells (53). During G1 to S phase progression, OGT is widely detected in the cytoplasm and nucleus, while as expected, cyclin D1 staining is mostly nuclear (Figure 3D). Indeed, upon mitogenic stimulation, cyclin D1 is rapidly transported into the nucleus together with its catalytic subunits CDK4/CDK6 (1, 6). PLA results indicate that the interaction between OGT and cyclin D1 can occur in both the cytoplasmic and nuclear compartments but is mainly detected in the nucleus of synchronized cycling cells (Figure 3E), suggesting that OGT is a new partner of the cyclin D1-CDK complex. Our study also unveils the potential *O*-GlcNAcylation of cyclin D1. Indeed, cyclin D1 was detected by Western blot following enrichment of *O*-GlcNAcylated proteins using either sWGA lectin-agarose beads or a click chemistry-based strategy (Figures 4B,C). *In situ* Duolink experiments using cyclin D1 and *O*-GlcNAc antibodies corroborate our findings (Figure 4A). However, it is unlikely that all the fluorescent spots detected by PLA are related to the *O*-GlcNAcylated forms of cyclin D1 since *O*-GlcNAcylation of cyclin D1 appears to occur at a low stoichiometry (Figures 4B,C). Given that cyclin D1 is mostly detected in the nucleus (Figure 3D), and that cyclin D1 and OGT preferentially interact in this compartment (Figure 3E), we hypothesize that OGT glycosylates the cyclin in the nucleus, which corresponds to a few nuclear spots that we observe by PLA (Figure 4A). The high PLA signal in the cytoplasm could arise from the binding of cyclin D1 to other potentially *O*-GlcNAcylated proteins with which the cyclin interacts (53). The chaperone Hsc70 could also be a good candidate, since it is highly *O*-GlcNAcylated (54) and promotes the stabilization of newly synthesized cyclin D1 (55). Thus, besides phosphorylation and ubiquitination, *O*-GlcNAcylation appears as an additional mean to finely regulate cyclin D1 turnover.

The effect of *O*-GlcNAcylation on cyclin D1 expression is in agreement with previous works showing that *O*-GlcNAcylation favors the stability of target proteins through the direct inhibition of their proteasomal degradation. *O*-GlcNAc modification can compete with a phosphorylation site that triggers ubiquitination, as previously shown for Ser10 of the transcription factor δ-lactoferrin (27) and Thr41 of β-catenin (47). One of our hypotheses is that OGT might directly impede phosphorylation at Thr286 by occupation of the residue by *O*-GlcNAc. The increase of *O*-GlcNAcylation could help in stabilizing cyclin D1 through a decrease of its GSK-3β-mediated phosphorylation at Thr286 which promotes its ubiquitin-mediated degradation (8, 9). *O*-GlcNAcylation can also compete with an adjacent phosphorylation site which promotes ubiquitination, as demonstrated for the tumor suppressor p53 for which *O*-GlcNAcylation at Ser149 impairs phosphorylation at Thr155 (44). Nine phosphorylation sites have been mapped on human cyclin D1 (Ser90, Ser111, Ser131, Ser197, Ser219, Tyr226, Ser234, Thr286, Thr288; see references in www.phosphosite.org, accession number P24385), but to date only Thr286 and Thr288 residues are known to have a biological effect once phosphorylated, both promoting the proteasomal degradation of cyclin D1 (8, 11, 12). Regarding the ubiquitination sites, over the 10 lysine residues that have been mapped on cyclin D1 (Lys 33, 46, 50, 95, 96, 114, 167, 175, 238, 269; see references in www.phosphosite.org), only Lys269 has been shown to be essential for ubiquitination and subsequent proteolysis of cyclin D1 (15). More experiments for mapping the *O*-GlcNAc site(s) of cyclin D1 are needed to further decipher whether *O*-GlcNAcylation of cyclin D1 competes or not with the phosphorylation or ubiquitination sites of cyclin D1, and thus helps in stabilizing the cyclin.

Other possible mechanism by which OGT may control cyclin D1 turnover is through the *O*-GlcNAcylation of GSK-3β since the kinase is itself *O*-GlcNAcylated (48, 56, 57). The negative regulation of GSK-3β activity by *O*-GlcNAcylation has been shown in HEK293FT cells and MKN45 gastric cancer cells but appears to be cell type-dependent (48, 56). Thiamet G-induced high *O*-GlcNAcylation of GSK3β might contribute to the decrease of cyclin D1 ubiquitination that we report in this study, as a consequence of a decrease in Thr286 phosphorylation. *O*-GlcNAcylation of GSK3β might also impair the activity of Fbxo4, the E3 ubiquitin ligase F-Box subunit which controls the proteasomal degradation of cyclin D1 (21). Indeed, the phosphorylation of Fbxo4 by GSK3β is necessary for its ubiquitin ligase activity (58). To ascertain these hypotheses, further experiments are needed to determine whether sustained *O*-GlcNAcylation of GSK-3β could be involved in the increase of cyclin D1 stability in MCF7 and HCT116 cancer cells, as shown for FoxM1 in gastric cancer cells (48).

On the other hand, OGT could modulate the ubiquitination of cyclin D1 by another ways. *O*-GlcNAc modification has been shown to directly regulate the proteasome activity. Several subunits of the proteasome are indeed *O*-GlcNAcylated (46) and it has been demonstrated that *O*-GlcNAcylation of Rpt2 ATPase in the 19S regulatory particle inhibits the proteolytic activity on some target proteins (52). Therefore, we cannot exclude the possibility that the decrease in cyclin D1 ubiquitination in ThG-treated cells results from a global inhibition of proteasome activity. More recently, fine regulation of the ubiquitin system by OGT has been evidenced. For example, the *O*-GlcNAcylation of the transcriptional cofactor YAP prevents its interaction with the E3 ubiquitin ligase subunit β-TRCP, thus decreasing its degradation (59). Conversely, *O*-GlcNAcylation of the transcriptional regulator PGC1α facilitates the binding of the deubiquitinase BAP1 to PGC1α, leading thus to its stabilization by blocking its ubiquitination (45). The SCF ubiquitin ligase is responsible for the polyubiquitination of cyclin D1. Different F-Box proteins are able to recognize cyclin D1, including Fbxo4, Fbxw8, Fbxo31, and Skp2 (13–16, 60). Cyclin D1 ubiquitination is reversed by the deubiquitinases USP2 and USP22 (17, 18). By using co-immunoprecipitation approaches, we tried to determine whether elevation of *O*-GlcNAcylation favors the binding of USP2 to cyclin D1, as described for BAP1 and PGC1α (45). Unfortunately, we could not conclude because we failed to detect the interaction of both proteins in HCT116 cells (data not shown), although it was previously reported in these cells (17). The potential regulation by OGT of either the ubiquitin ligases or deubiquitinases that control cyclin D1 turnover might also explain how *O*-GlcNAc modification takes part in cyclin D1 homeostasis. Future work would decipher the exact molecular mechanisms underlying the modulation of cyclin D1 ubiquitination by OGT.

To conclude, this study provides new insights into the positive regulation of cyclin D1 stability by *O*-GlcNAcylation and opens new perspectives on the contribution of hyper-*O*-GlcNAcylation to the deregulation of cell proliferation in cancer.

## Author Contributions

LM, VD, TL, and A-SV-E: designed the experiments; LM, VD, MM, CS, ML, and A-SV-E: performed the experiments; LM, VD, and A-SV-E: analyzed the data; A-SV-E: wrote the manuscript; LM, VD, and TL: edited the manuscript. All the authors approved the manuscript.

### Conflict of Interest Statement

The authors declare that the research was conducted in the absence of any commercial or financial relationships that could be construed as a potential conflict of interest.

## References

[1] Massagué J. G1 cell-cycle control and cancer. Nature (2004) 432:298–306. 10.1038/nature03094 15549091

[2] Qie S Diehl JA. Cyclin D1, cancer progression, and opportunities in cancer treatment. J Mol Med. (2016) 94:1313–26. 10.1007/s00109-016-1475-3 27695879 PMC5145738

[3] Lavoie JN L'Allemain G Brunet A Müller R Pouysségur J. Cyclin D1 expression is regulated positively by the p42/p44MAPK and negatively by the p38/HOGMAPK pathway. J Biol Chem. (1996) 271:20608–16. 8702807 10.1074/jbc.271.34.20608

[4] Aktas H Cai H Cooper GM Ras links growth factor signaling to the cell cycle machinery via regulation of cyclin D1 and the cdk inhibitor p27 Kip1. Mol Cell Biol. (1997) 17: 3850–57. 10.1128/MCB.17.7.3850 9199319 PMC232237

[5] Muise-Helmericks RC Grimes HL Bellacosa A Malstrom SE Tsichlis PN Rosen N. Cyclin D expression is controlled post-transcriptionally via a phosphatidylinositol 3-kinase/Akt-dependent pathway. J Biol Chem. (1998) 273:29864–72. 10.1074/jbc.273.45.29864 9792703

[6] Baldin V Lukas J Marcote MJ Pagano M Draetta G. Cyclin D1 is a nuclear protein required for cell cycle progression in G1. Genes Dev. (1993) 7:812–21. 10.1101/gad.7.5.812 8491378

[7] Chen H Xu X Wang G Zhang B Wang G Xin G. CDK4 protein is degraded by anaphase-promoting complex/cyclosome in mitosis and reaccumulates in early G1 phase to initiate a new cell cycle in HeLa cells. J Biol Chem. (2017) 292:10131–41. 10.1074/jbc.M116.773226. 28446612 PMC5473219

[8] Diehl JA Zindy F Sherr CJ. Inhibition of cyclin D1 phosphorylation on threonine-286 prevents its rapid degradation via the ubiquitin-proteasome pathway. Genes Dev. (1997) 11:957–72. 10.1101/gad.11.8.957 9136925

[9] Guo Y Yang K Harwalkar J Nye JM Mason DR Garrett MD. Phosphorylation of cyclin D1 at Thr 286 during S phase leads to its proteasomal degradation and allows efficient DNA synthesis Oncogene (2005) 24:2599–612. 10.1038/sj.onc.1208326 15735756

[10] Alt JR Cleveland JL Hannink M Diehl JA. Phosphorylation-dependent regulation of cyclin D1 nuclear export and cyclin D1-dependent cellular transformation. Genes Dev. (2000) 14: 3102–14. 10.1101/gad.854900 11124803 PMC317128

[11] Benzeno S Diehl JA. C-terminal sequences direct cyclin D1-CRM1 binding. J Biol Chem. (2004) 279:56061–6. 10.1074/jbc.M411910200 15513923

[12] Zou Y Ewton DZ Deng X Mercer SE Friedman E. Mirk/dyrk1B kinase destabilizes cyclin D1 by phosphorylation at threonine 288. J Biol Chem. (2004) 279:27790–8. 10.1074/jbc.M403042200 15075324

[13] Lin DI Barbash O Kumar KG Weber JD Harper JW Klein-Szanto AJ. Phosphorylation-dependent ubiquitination of cyclin D1 by the SCF(FBX4-alphaB crystallin) complex. Mol Cell. (2006) 24:355–66. 10.1016/j.molcel.2006.09.007 17081987 PMC1702390

[14] Okabe H Lee SH Phuchareon J Albertson DG McCormick F Tetsu O. A critical role for FBXW8 and MAPK in cyclin D1 degradation and cancer cell proliferation. PLoS ONE (2006) 1:e128. 10.1371/journal.pone.0000128 17205132 PMC1762433

[15] Barbash O Egan E Pontano LL Kosak J Diehl JA. Lysine 269 is essential for cyclin D1 ubiquitylation by the SCF(Fbx4/alphaB-crystallin) ligase and subsequent proteasome-dependent degradation. Oncogene (2009) 28:4317–25. 10.1038/onc.2009.287. 19767775 PMC2794935

[16] Kanie T Onoyama I Matsumoto A Yamada M Nakatsumi H Tateishi Y. Genetic reevaluation of the role of F-box proteins in cyclin D1 degradation. Mol Cell Biol. (2012) 32:590–605. 10.1128/MCB.06570-11. 22124152 PMC3266600

[17] Shan J Zhao W Gu W. Suppression of cancer cell growth by promoting cyclin D1 degradation. Mol Cell. (2009) 36:469–76. 10.1016/j.molcel.2009.10.018 19917254 PMC2856324

[18] Gennaro VJ Stanek TJ Peck AR Sun Y Wang F Qie S. Control of CCND1 ubiquitylation by the catalytic SAGA subunit USP22 is essential for cell cycle progression through G1 in cancer cells. Proc Natl Acad Sci USA. (2018) 115:E9298–307. 10.1073/pnas.1807704115 30224477 PMC6176615

[19] Davis MI Pragani R Fox JT Shen M Parmar K Gaudiano EF. Small molecule inhibition of the ubiquitin-specific protease USP2 accelerates cyclin D1 degradation and leads to cell cycle arrest in colorectal cancer and mantle cell lymphoma models. J Biol Chem. 2016 291:24628–40. 10.1074/jbc.M116.738567 27681596 PMC5114414

[20] Lu F Gladden AB Diehl JA. An alternatively spliced cyclin D1 isoform, cyclin D1b, is a nuclear oncogene. Cancer Res. (2003) 63: 7056–61. 14612495

[21] Barbash O Zamfirova P Lin DI Chen X Yang K Nakagawa H. Mutations in Fbx4 inhibit dimerization of the SCF(Fbx4) ligase and contribute to cyclin D1 overexpression in human cancer. Cancer Cell (2008) 14:68–78. 10.1016/j.ccr.2008.05.017 18598945 PMC2597358

[22] Tan EP Duncan FE Slawson C. The sweet side of the cell cycle. Biochem Soc Trans. (2017) 45:313–22. 10.1042/BST20160145 28408472 PMC5515282

[23] Bond MR Hanover JA. *O*-GlcNAc cycling: a link between metabolism and chronic disease. Annu Rev Nutr. (2013) 33:205–29. 10.1146/annurev-nutr-071812-161240. 23642195 PMC10483992

[24] Hardivillé S Hart GW. Nutrient regulation of signaling, transcription, and cell physiology by O-GlcNAcylation. Cell Metab. (2014) 20:208–13. 10.1016/j.cmet.2014.07.014 25100062 PMC4159757

[25] Nagel AK Ball LE. Intracellular protein O-GlcNAc modification integrates nutrient status with transcriptional and metabolic regulation. Adv Cancer Res. (2015) 126:137–66. 10.1016/bs.acr.2014.12.003 25727147

[26] Chou TY Hart GW Dang CV. c-Myc is glycosylated at threonine 58, a known phosphorylation site and a mutational hot spot in lymphomas. J Biol Chem. (1995) 270:18961–5. 10.1074/jbc.270.32.18961 7642555

[27] Hardivillé S Hoedt E Mariller C Benaïssa M Pierce A. O-GlcNAcylation/phosphorylation cycling at Ser10 controls both transcriptional activity and stability of delta-lactoferrin. J Biol Chem. (2010) 285:19205–18. 10.1074/jbc.M109.080572 20404350 PMC2885199

[28] Luo B Soesanto Y McClain DA. Protein modification by O-Linked GlcNAc reduces angiogenesis by inhibiting Akt activity in endothelial cells. Arterioscler Thromb Vasc Biol. (2008) 28:651–7. 10.1161/ATVBAHA.107.159533 18174452 PMC2734484

[29] Ferrer CM Sodi VL Reginato MJ. O-GlcNAcylation in Cancer biology: linking metabolism and signaling. J Mol Biol. (2016) 428:3282–94. 10.1016/j.jmb.2016.05.028 27343361 PMC4983259

[30] Chiaradonna F Ricciardiello F Palorini R. The nutrient-sensing hexosamine biosynthetic pathway as the hub of cancer metabolic rewiring. Cells (2018) 7:E53. 10.3390/cells7060053 29865240 PMC6025041

[31] Slawson C Zachara NE Vosseller K Cheung WD Lane MD Hart GW. Perturbations in O-linked beta-N-acetylglucosamine protein modification cause severe defects in mitotic progression and cytokinesis. J Biol Chem. (2005) 280:32944–56. 10.1074/jbc.M503396200 16027160

[32] Sakabe K Hart GW. O-GlcNAc transferase regulates mitotic chromatin dynamics. J Biol Chem. (2010) 285:34460–68. 10.1074/jbc.M110.158170 20805223 PMC2966060

[33] Drougat L Olivier-Van Stichelen S Mortuaire M Foulquier F Lacoste AS Michalski JC. Characterization of O-GlcNAc cycling and proteomic identification of differentially O-GlcNAcylated proteins during G1/S transition. Biochim Biophys Acta (2012) 1820:1839–48. 10.1016/j.bbagen.2012.08.024 22967762

[34] Olivier-Van Stichelen S Drougat L Dehennaut V El Yazidi-Belkoura I Guinez C Mir AM Serum-stimulated cell cycle entry promotes ncOGT synthesis required for cyclin D expression. Oncogenesis (2012) 1:e36 10.1038/oncsis.2012.36 23552487 PMC3545199

[35] O'Donnell N Zachara NE Hart GW Marth JD. Ogt-dependent X-chromosome-linked protein glycosylation is a requisite modification in somatic cell function and embryo viability. Mol Cell Biol. (2004) 24:1680–90. 10.1128/MCB.24.4.1680-1690.2004 14749383 PMC344186

[36] Caldwell SA Jackson SR Shahriari KS Lynch TP Sethi G Walker S. Nutrient sensor O-GlcNAc transferase regulates breast cancer tumorigenesis through targeting of the oncogenic transcription factor FoxM1. Oncogene (2010) 29:2831–42. 10.1038/onc.2010.41 20190804

[37] Lynch TP Ferrer CM Jackson SR Shahriari KS Vosseller K Reginato MJ. Critical role of O-Linked β-N-acetylglucosamine transferase in prostate cancer invasion, angiogenesis, and metastasis. J Biol Chem. (2012) 287:11070–81. 10.1074/jbc.M111.302547 22275356 PMC3322861

[38] Ma Z Vocadlo DJ Vosseller K. Hyper-O-GlcNAcylation is anti-apoptotic and maintains constitutive NF-κB activity in pancreatic cancer cells. J Biol Chem. (2013) 288:15121–30. 10.1074/jbc.M113.470047 23592772 PMC3663532

[39] Lanza C Tan EP Zhang Z Machacek M Brinker AE Azuma M. Reduced O-GlcNAcase expression promotes mitotic errors and spindle defects. Cell Cycle (2016) 15:1363–75. 10.1080/15384101.2016.1167297 27070276 PMC4889296

[40] Li J Deng M Wei Q Liu T Tong X Ye X. Phosphorylation of MCM3 protein by cyclin E/cyclin-dependent kinase 2 (Cdk2) regulates its function in cell cycle. J Biol Chem. (2011) 286:39776–85. 10.1074/jbc.M111.226464 21965652 PMC3220541

[41] Leturcq M Mortuaire M Hardivillé S Schulz C Lefebvre T Vercoutter-Edouart AS. *O*-GlcNAc transferase associates with the MCM2-7 complex and its silencing destabilizes MCM-MCM interactions. Cell Mol Life Sci. (2018) 75:4321–39. 10.1007/s00018-018-2874-0 30069701 PMC6208770

[42] Dehennaut V Slomianny MC Page A Vercoutter-Edouart AS Jessus C Michalski JC. Identification of structural and functional O-linked N-acetylglucosamine-bearing proteins in *Xenopus laevis* oocyte. Mol Cell Proteom. (2008) 7:2229–45. 10.1074/mcp.M700494-MCP200 18617508

[43] Guo Y Harwalkar J Stacey DW Hitomi M. Destabilization of cyclin D1 message plays a critical role in cell cycle exit upon mitogen withdrawal. Oncogene (2005) 24:1032–42. 10.1038/sj.onc.1208299 15592507

[44] Yang W Kim H Nam JE Ju HW Kim JW Kim HS. Modification of p53 with O-linked N-acetylglucosamine regulates p53 activity and stability. Nat Cell Biol. (2006) 8: 1074–83. 10.1038/ncb1470 16964247

[45] Ruan HB Han X Li MD Singh JP Qian K Azarhoush S. O-GlcNAc transferase/host cell factor C1 complex regulates gluconeogenesis by modulating PGC-1α stability. Cell Metab. (2012) 16:226–37. 10.1016/j.cmet.2012.07.006 22883232 PMC3480732

[46] Ruan HB Nie Y Yang X. Regulation of protein degradation by O-GlcNAcylation: crosstalk with ubiquitination. Mol Cell Proteom. (2013) 12:3489–97. 10.1074/mcp.R113.029751 23824911 PMC3861702

[47] Olivier-Van Stichelen S Dehennaut V Buzy A Zachayus JL Guinez C Mir AM O-GlcNAcylation stabilizes β-catenin through direct competition with phosphorylation at threonine 41. Faseb J. (2014) 28:3325–38. 10.1096/fj.13-243535 24744147 PMC4101651

[48] Inoue Y Moriwaki K Ueda Y Takeuchi T Higuchi K Asahi M. Elevated O-GlcNAcylation stabilizes FOXM1 by its reduced degradation through GSK-3β inactivation in a human gastric carcinoma cell line, MKN45 cells. Biochem Biophys Res Commun. (2018) 495:1681–87. 10.1016/j.bbrc.2017.11.179 29196265

[49] Whisenhunt TR Yang X Bowe DB Paterson AJ Van Tine BA Kudlow JE. Disrupting the enzyme complex regulating O-GlcNAcylation blocks signaling and development. Glycobiology (2006) 16:551–63. 10.1093/glycob/cwj096 16505006

[50] Jarvius M Paulsson J Weibrecht I Leuchowius KJ Andersson AC Wählby C. *In situ* detection of phosphorylated platelet-derived growth factor receptor b using a generalized proximity ligation method. Mol Cell Proteom. (2007) 6:1500–09. 10.1074/mcp.M700166-MCP200 17565975

[51] Conze T Carvalho AS Landegren U Almeida R Reis CA David L. MUC2 mucin is a major carrier of the cancer-associated sialyl-Tn antigen in intestinal metaplasia and gastric carcinomas. Glycobiology (2010) 20:199–206. 10.1093/glycob/cwp161 19815850

[52] Zhang F Su K Yang X Bowe DB Paterson AJ Kudlow JE. O-GlcNAc modification is an endogenous inhibitor of the proteasome. Cell (2003) 115:715–25. 10.1016/S0092-8674(03)00974-7 14675536

[53] Jirawatnotai S Hu Y Michowski W Elias JE Becks L Bienvenu F. A function for cyclin D1 in DNA repair uncovered by protein interactome analyses in human cancers. Nature (2011) 474:230–4. 10.1038/nature10155 21654808 PMC3134411

[54] Guinez C Losfeld ME Cacan R Michalski JC Lefebvre T. Modulation of HSP70 GlcNAc-directed lectin activity by glucose availability and utilization. Glycobiology (2006) 16:22–8. 10.1093/glycob/cwj041 16177265

[55] Diehl JA Yang W Rimerman RA Xiao H Emili A. Hsc70 regulates accumulation of cyclin D1 and cyclin D1-dependent protein kinase. Mol Cell Biol. (2003) 23:1764–74. 10.1128/MCB.23.5.1764-1774.2003 12588994 PMC151693

[56] Shi J Wu S Dai CL Li Y Grundke-Iqbal I Iqbal K. Diverse regulation of AKT and GSK-3β by O-GlcNAcylation in various types of cells. FEBS Lett. (2012) 586:2443–50. 10.1016/j.febslet.2012.05.063 22687243 PMC3407308

[57] Maury JJ Ng D Bi X Bardor M Choo AB. Multiple reaction monitoring mass spectrometry for the discovery and quantification of O-GlcNAc-modified proteins. Anal Chem. (2014) 86:395–402. 10.1021/ac401821d 24144119

[58] Barbash O Diehl JA. SCF (Fbx4/alphaB-crystallin) E3 ligase: when one is not enough. Cell Cycle (2008) 7:2983–86. 10.4161/cc.7.19.6775 18818515

[59] Zhang X Qiao Y Wu Q Chen Y Zou S Liu X. The essential role of YAP O-GlcNAcylation in high-glucose-stimulated liver tumorigenesis. Nat Commun. (2017) 8:15280. 10.1038/ncomms15280 28474680 PMC5424161

[60] Li Y Jin K Bunker E Zhang X Luo X Liu X. Structural basis of the phosphorylation-independent recognition of cyclin D1 by the SCF^FBXO31^ ubiquitin ligase. Proc Natl Acad Sci USA. (2018) 115:319–24. 10.1073/pnas.1708677115 29279382 PMC5777030

